# A Cell-Free Translocation System Using Extracts of Cultured Insect Cells to Yield Functional Membrane Proteins

**DOI:** 10.1371/journal.pone.0112874

**Published:** 2014-12-08

**Authors:** Toru Ezure, Kei Nanatani, Yoko Sato, Satomi Suzuki, Keishi Aizawa, Satoshi Souma, Masaaki Ito, Takahiro Hohsaka, Gunnar von Heijine, Toshihiko Utsumi, Keietsu Abe, Eiji Ando, Nobuyuki Uozumi

**Affiliations:** 1 Department of Molecular Engineering, Graduate School of Engineering, Tohoku University, Sendai, Japan; 2 Clinical and Biotechnology B.U., Shimadzu Corporation, Kyoto, Japan; 3 Department of Microbial Biotechnology, Graduate School of Agricultural Science, Tohoku University, Sendai, Japan; 4 School of Materials Science, Japan Advanced Institute of Science and Technology, Ishikawa, Japan; 5 Department of Biochemistry and Biophysics, Stockholm University, Stockholm, Sweden; 6 Department of Biological Chemistry, Faculty of Agriculture, Yamaguchi University, Yamaguchi, Japan; University of Saskatchewan, Canada

## Abstract

Cell-free protein synthesis is a powerful method to explore the structure and function of membrane proteins and to analyze the targeting and translocation of proteins across the ER membrane. Developing a cell-free system based on cultured cells for the synthesis of membrane proteins could provide a highly reproducible alternative to the use of tissues from living animals. We isolated Sf21 microsomes from cultured insect cells by a simplified isolation procedure and evaluated the performance of the translocation system in combination with a cell-free translation system originating from the same source. The isolated microsomes contained the basic translocation machinery for polytopic membrane proteins including SRP-dependent targeting components, translocation channel (translocon)-dependent translocation, and the apparatus for signal peptide cleavage and N-linked glycosylation. A transporter protein synthesized with the cell-free system could be functionally reconstituted into a lipid bilayer. In addition, single and double labeling with non-natural amino acids could be achieved at both the lumen side and the cytosolic side in this system. Moreover, tail-anchored proteins, which are post-translationally integrated by the guided entry of tail-anchored proteins (GET) machinery, were inserted correctly into the microsomes. These results showed that the newly developed cell-free translocation system derived from cultured insect cells is a practical tool for the biogenesis of properly folded polytopic membrane proteins as well as tail-anchored proteins.

## Introduction

Membrane proteins constitute almost one third of all gene products in any type of organism. Because membrane proteins are embedded in the cell membrane they are in direct contact with the outside of the cell and are major targets for pharmaceutical or physiological regulation. For this reason there is frequently a need to be able to produce these proteins in the laboratory. Commonly, this is done by expression in a heterologous system like *E. coli*. However, overexpression of foreign proteins often leads to cytosolic toxicity in the host cells, resulting in a failure to produce sufficient amounts of protein. In *vitro* synthesis of membrane proteins is an alternative method to overcome the problems encountered with heterologous systems. A cell-free translation/translocation system is the preferred method to expedite the production of a membrane protein of interest. In eukaryotes, most membrane proteins are co-translationally inserted into the membrane of the rough endoplasmic reticulum (ER), assisted by the secretion machinery involving the translocon [1]. Recently, a novel protein-targeting pathway, the guided entry of tail anchored proteins (GET) pathway, that directs the targeting machinery for tail-anchored membrane proteins (TA-proteins) to the ER membrane, has been described [2]. This targeting process occurs post-translationally, since TA-proteins have no signal peptide at the N-terminus and contain a single transmembrane domain at the C-terminus. In order to deliver functional membrane proteins to the ER membrane, it is necessary that a cell-free translation/translocation system preserve the integrity of the involved pathways.

Several types of cell-free translation systems have been developed from *E. coli* [3], [4], yeast [5], wheat germ [6], [7], rabbit reticulocytes [8] or insect cells [9], [10]. For the cell-free translocation of eukaryotic membrane proteins and secreted proteins, rough microsomes isolated from dog pancreas [11] and *Drosophila* S2 cells [12] are reported; in particular, the former dog pancreas system is also commercially available as a kit. By combining dog pancreas rough microsomes with rabbit reticulocytes, synthesis of membrane proteins can be achieved in a single tube. This established cell-free cotranslational membrane protein translocation system based on animal cells has been widely used for the analysis of the mechanism of translocation and integration of proteins into the lipid bilayer. However, the quality of cell lysate and microsomes can be inconsistent since it depends on the state of the animal from which the starting materials were harvested. To overcome this limitation, a cell-free translation system based on cultured insect cells has been developed for the synthesis of soluble protein; it is also available as a commercial kit (Transdirect *insect cell*; Shimadzu, Kyoto, Japan) [9], [10]. Cultured insect cells, e.g., *Spodoptera frugiperda* 21 (Sf21) cells can be readily grown in large scale fermenter cultures without the necessity to sacrifice animals. In order to adapt the system for the synthesis of membrane proteins, microsomes containing the protein translocation machinery have to be included because proper folding of membrane proteins occurs in the ER membrane.

**Figure 1:**
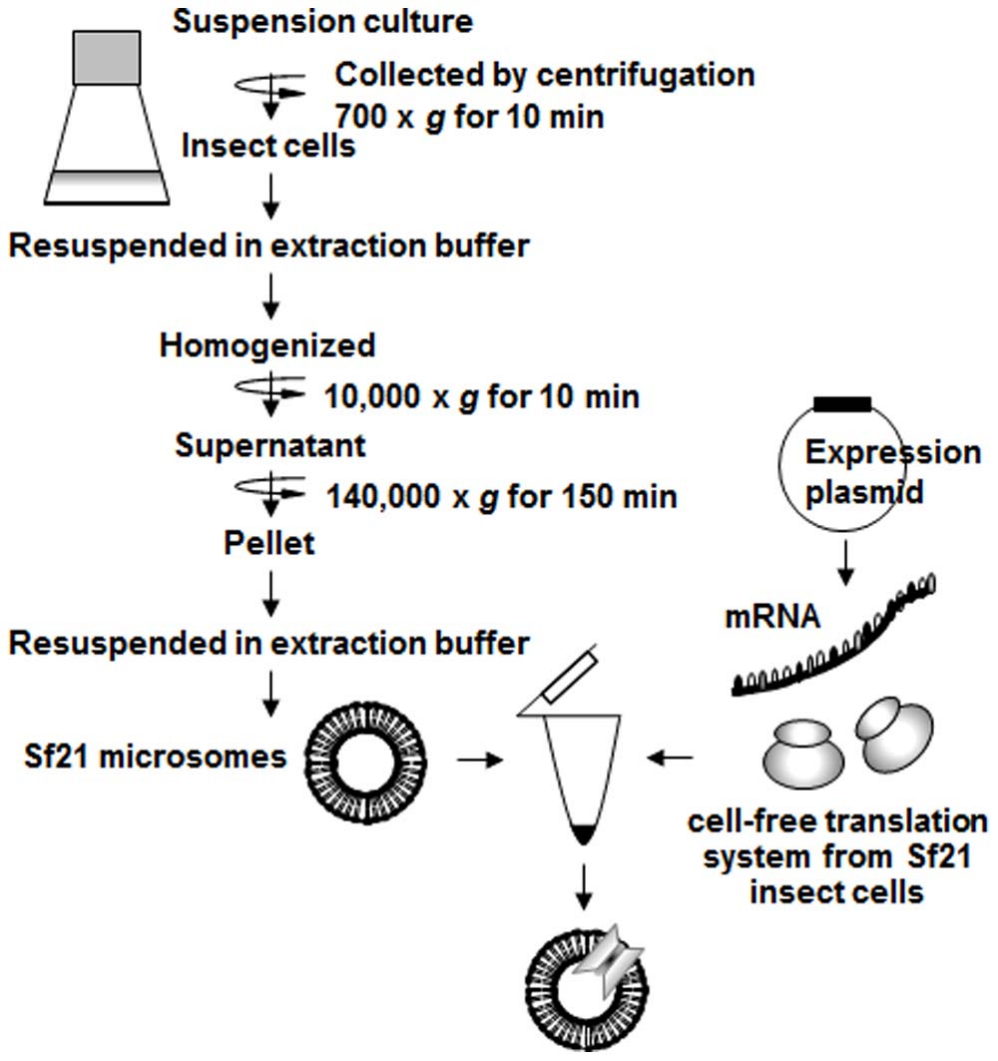
Flow chart for the preparation of microsomes from Sf21 cultured insect cells. Fewer centrifugation and wash steps were required compared to the conventional protocol for the preparation of rough microsomes from dog pancreas and the composition of the buffer was simplified. Single-tube reactions consisting of Sf21microsomes, cell-free translationally active lysates from cultured insect cells and mRNA were performed to synthesize membrane proteins *in vitro*.

Here we examine the use of ER membranes from Sf21 cultured insect cells as a novel translocation system for membrane protein synthesis (Fig. 1). Production of several examples of membrane proteins and their correct post-translational modification were tested using this system. Our results demonstrate that this cell-free translocation system derived from cultured insect cells can be used as a reliable tool to enable the highly reproducible production of membrane proteins *in vitro*. In addition, the cell-free system made it possible to incorporate non-natural amino acids with artificial groups such as fluorophores into membrane proteins in a position-specific manner. In summary, the methods presented here provide a new tool that will be useful for understanding the structure and function of membrane proteins in general.

## Materials and Methods

### Preparation of rough microsomes (Sf21 microsomes) from cultured insect cells

For the isolation of extracts containing the translational machinery from cultured *Spodoptera frugiperda* 21 (Sf21) cells a modified method based on a method for isolation of rough microsomes from dog pancreas was used [13], [14]. Sf21 insect cells (Invitrogen, San Diego, CA) were grown in suspension culture at 27°C in Sf-900 II serum-free medium (GIBCO, Grand Island, NY). The insect cells were harvested by centrifugation for 10 min at 700×*g* at 4°C. The cell pellets were suspended in buffer A (40 mM HEPES-KOH (pH 7.9), 250 mM sucrose and 1 mM DTT) at approximately 0.33 g/ml. This suspension was homogenized in a Dounce homogenizer (20 strokes). The ruptured cells were removed by centrifugation for 10 min at 10,000×*g* at 4°C. The supernatant was layered over buffer B (40 mM HEPES-KOH (pH 7.9), 1.3 M sucrose and 1 mM DTT) at a ratio of supernatant to buffer B at approximately 3∶1 and centrifuged for 150 min at 140,000×*g* at 4°C. Afterwards the remaining upper solution and buffer B were removed and the pellet was resuspended in buffer A by homogenization with a syringe (Ø 0.3 mm). The solution was adjusted to an OD_280_ of 100 to 250 with buffer B, and stored at −80°C.

### Plasmid constructs and RNA synthesis

The genes encoding *E. coli* β-lactamase, human pro-tumor necrosis factor (pro-TNF) [15], human synaptobrevin 2 (Syb2), *E. coli* Na^+^/H^+^ antiporter (NhaA), *Tetragenococcus halophilus* aspartate: alanine antiporter (AspT), *Aeropyrum pernix* K^+^ channel (KvAP), *Arabidopsis thaliana* K^+^ channels (AtKC1, KAT1, KAT2, GORK and AKT1), *E. coli* ABC transporter (MsbA) or human Sec61β were cloned into the multi-cloning site of pTD1 (accession no. AB194742) or pTD1-strep [16], to be expressed under control of the T7 promoter (Table 1). To construct the plasmid containing β-lactamase lacking its signal peptide sequence (SIQHFRVALIPFFAAFCLPVFA), designated as Δsp- β-lactamase, a primer set of 5'-ATAAAATATAAAGATATGCACCCAGAAACGCTGG-3' and 5'-GCCGCCCGACTCTAGATTACTTTTCAAACTGCGGATGG-3' were used. Some constructs also contained an added His tag, prolactin or strep-tag II to enable immunological protein detection (data not shown; Table 1). In order to introduce an N-type glycosylation site into KvAP, the “g-loop” sequence (NXS/T) was inserted at the extracellular loop between transmembrane segments (TMS) S3 and S4. The C-terminal extracellular region following the single TMS of the TA-proteins Syb2 and Sec61β was swapped with a sequence containing a glycosylation acceptor site (GVPYVSSSDSGSGGGNKNITQAPPH) to enable detection of their translocation [17], [18], [19]. The construct of pro-TNF has been described previously [20], [21]; it contains an added glycosylation site in the external C-terminal region. Site-directed mutagenesis was performed using the QuikChange mutagenesis kit (Stratagene). Amplified DNA sequences were verified by DNA sequencing. For the synthesis of mRNAs for cell-free protein synthesis, the template DNA was amplified by PCR with 5'-GCAGATTGTACTGAGAGTG-3' and 5'-GGAAACAGCTATGACCATG-3' as primers. The amplified DNA was purified by ethanol precipitation followed by phenol-chloroform extraction and used as a template for mRNA synthesis with the RiboMAX Large Scale RNA Production System-T7 (Promega) as described previously [10].

**Table 1:** List of constructs used.

figure	name	gene	source	No. TMS	insertion machinery	modifications	vector
Fig.2	β-lactamase	β-lactamase	*E. coli*	None (secreted)	translocon	Strep-tag sequence fused to the C-terminus of β-lactamase	pTD1-strep
	KvAP	K^+^ channel	*Aeropyrum pernix*	6		prolactin containing g-loop fused to cytosolic C-terminus of KvAP. G-loop at extracellular loop between S3 and S4	pTD1
	pro-TNF	pro-tumor necrosis factor	human	1		N-glycosylation site at the extracellular C-terminal region	pTD1
	Syb2	synaptobrevin II	human	1	GET	Drosophila-based GVPYVSSSDSGSGGGNKNITQAPPH sequence containing engineered glycosylation site at C terminal region after transmembrane of Syb2	pTD1
Fig.3	AtKC1	K^+^ channel	*Arabidopsis thaliana*	6	translocon	Strep-tag sequence fused to the C-terminus of each protein	pTD1-strep
	KAT1						pTD1-strep
	KAT2						pTD1-strep
	GORK						pTD1-strep
	AKT1						pTD1-strep
	NhaA	Na^+^/H^+^ antiporter	*E. coli*	12		Strep-tag sequence fused to the C-terminus of NhaA.	pTD1-strep
	AspT	aspartate: alanine antiporter	*Tetragenococcus halophilus*	10		6× His tag sequence at the C-terminus	pTD1
	MsbA	ABC transporter	*E. coli*	6		6× His tag sequence at the N-terminus	pTD1
	Sec61β	Sec61β	human	1	GET	Drosophila-based GVPYVSSSDSGSGGGNKNITQAPPH sequence containing engineered glycosylation site at C terminal region after transmembrane of Sec61β	pTD1
Fig. 4	AspT-His	AspT-His	*Tetragenococcus halophilus*	10	translocon	6× His tag at a cytoplasmic large loop between the 5th and the 6th transmembrane domain	pTD1
Fig. 5A, 5B	NhaA	Na^+^/H^+^ antiporter	*E. coli*	12	translocon	8× His tag sequence at the C-terminus	pTD1
Fig. 5C	NhaA	Na^+^/H^+^ antiporter	*E. coli*	12	translocon	Addition of Gly-Gly, non-natural amino acid immediately after V388 in the NhaA construct as shown in Fig. 3.	pTD1-strep
Fig. 6	KvAP	K^+^ channel	*Aeropyrum pernix*	6	translocon	See Fig. 2.	pTD1
	pro-TNF	pro-tumor necrosis factor	human	1		See Fig. 2.	pTD1
	Syb2	synaptobrevin 2	human	1	GET	See Fig. 2.	pTD1

### Cell-free protein synthesis

Cell-free protein synthesis was carried out using the Transdirect *insect cell* (Shimadzu, Kyoto, Japan) and cell-free protein translation system prepared from Sf21 insect cells according to the instruction manual. Sf21 microsomes were added to the reaction mixture at a final concentration of 8% (v/v). Radioisotope labeling of the synthesized proteins was performed by addition of ^35^S-methionine (Muromachi Yakuhin Co., Tokyo, Japan) to a final concentration of 0.55 mM. After the synthesis reaction, samples were subjected to SDS-PAGE (10% or 15% polyacrylamide). Gels were dried and exposed to an imaging plate, which was analyzed with a Fujifilm FLA-7000 image reader (FUJIFILM Life Science, Tokyo, Japan). For the synthesis of fluorescently labeled proteins, 1 µl of FluoroTect Green_Lys_ tRNA (Promega, Madison, WI) was added to 50 µl of reaction mixture. BODIPY FL-AF-tRNA_CUA_, BODIPY 558-AF-tRNA_CUA_, IC3-AF-tRNA_CUA_, IC5-AF-tRNA_CUA_, and BODIPY FL-AF-tRNA_CCCG_ were synthesized as described [22], [23]. Position-specific incorporation of fluorescent non-natural amino acids was performed through addition of TAMRA-AF-tRNA_CUA_ (ProteinExpress, Chiba, Japan), BODIPY FL-AF-tRNA_CUA_, BODIPY 558-AF-tRNA_CUA_, IC3-AF-tRNA_CUA_, IC5-AF-tRNA_CUA_ or BODIPY FL-AF-tRNA_CCCG_ at a final concentration of 6.4 µM. For the synthesis of double-labeled NhaA, BODIPY 558-AF-tRNA_CUA_ and BODIPY FL-AF-tRNA_CCCG_ were added to the reaction mixture at a final concentration of 6.4 µM each. All reactions were carried out for 4 h at 25°C. The signal on the SDS-PAGE gel was detected with a laser-based fluorescent imager (Molecular Imager FX; Bio-Rad, Hercules, CA), as described previously [24].

### Purification and reconstitution of the AspT aspartate transporter

AspT was expressed as a 6× His-tagged protein, with the tag inserted into the large cytoplasmic loop between the 5th and 6th transmembrane domain [25]. After cell-free synthesis of AspT-His (10 ml reaction), microsomes were collected by centrifugation at 134,000×*g* for 30 min at 4°C. Solubilization and purification of AspT-His were performed as previously described [26], [27]. Briefly, microsomes were solubilized at 4°C for 2 h with 1 ml of 20 mM potassium phosphate buffer (pH 7) containing 1.5% (w/v) n-dodecyl-β-D-maltoside (DDM) (Nacalai, Kyoto, Japan), 50 mM L-aspartate and 20% glycerol. After centrifugation at 134,000×*g* for 30 min, the supernatant was incubated with 50 µL TALON Metal Affinity Resin (Clontech) at 4°C for 2 h. The resin was transferred to a column (Ultrafree-MC-GV Centrifugal Filters Durapore-PVDF 0.22 µm, BioRad) and the column was washed on ice with 1 ml of 20 mM potassium phosphate buffer (pH 7) containing 0.01% (w/v) DDM, 50 mM L-aspartate and 20% glycerol. AspT-His was eluted from the resin by brief centrifugation with 50 µl of wash buffer supplemented with 0.25 M imidazole. The concentration of AspT-His in the eluted fraction was determined and the sample was stored at −80°C. Isolation of AspT-His expressed in *E. coli* to be used as a control was performed as described previously (24). For reconstitution of AspT-His an aliquot of the eluted fraction containing 2.5 µg of AspT-His was diluted with the elution buffer to approximately 800 µl volume. After adding 130 µl of bath-sonicated *E. coli* phospholipids (6.5 µg, Avanti Polar Lipids, Alabaster, AL) and 18 µl of 15% 1-*O*-n-octyl- *β-*D-glucopyranoside (Nacalai, Kyoto, Japan) the volume was adjusted to 1 ml with elution buffer. After incubation for 20 min on ice, the mixture was rapidly injected into 20 ml of a solution containing 100 mM phosphate buffer (pH 7) supplemented with 100 mM potassium L-aspartate. The L-aspartate-loaded proteoliposomes were kept at 25°C for 20 min and then collected by centrifugation at 134,000×*g* for 30 min at 4°C.

### Aspartate transport assay

L-aspartate transport activity of AspT was measured as described previously [26], [27]. Concentrated proteoliposomes with reconstituted AspT were diluted with 50 mM potassium phosphate buffer (pH 7) containing 100 mM K_2_SO_4_. After 3 min of pre-incubation at 25°C, L-[2,3-^3^H]-aspartate (GE Healthcare, Piscataway, NJ) was added to a final concentration of 150 µM. At the indicated times, proteoliposomes (50 µl samples) were collected by vacuum filtration (0.22- µm-pore-size GSTF filter, Millipore, Billerica, MA), washed twice with 3 mL of the same potassium phosphate buffer and radioactivity was determined by scintillation counting. For the quantification of AspT-His, proteoliposomes containing AspT-His were subjected to SDS-PAGE on Criterion TGX Precast Gels (10%) (Bio-Rad) at 4°C. AspT-His purified from *E. coli* (0–20 ng) was used as a standard on the same gel. After electrophoresis, the gel was stained with Lumitein staining solution (BIOTIUM) in the dark. The intensity of the bands was quantitated with help of a PharosFX Molecular Imager with Quantity One software (Bio-Rad) and Image J.

## Results

### Isolation of rough microsomes from Sf21 cells

To prepare microsomal extracts containing the translational machinery from Sf21 cultured insect cells, we modified a procedure developed for the isolation of dog pancreas rough microsomes (Fig. 1 and S1 Figure) [1], [13], [14]. Since cultured cells are different from tissues and organs such as dog pancreas, the total number of centrifugation steps could be reduced. The “high-ionic-strength buffers” described for isolation of dog pancreas rough microsomes were not required [13]. The buffer solution used to suspend the Sf21 cell extract contained sucrose and DTT in HEPES-KOH, without the need for added nucleases or metal ions. This simplified buffer used for the microsome preparation was adjusted to be similar to the solution used for the translation reaction, thereby making it less likely to cause inhibition of the translation step.

### Evaluation of posttranslational modification activity of Sf21 microsomes

**Figure 2:**
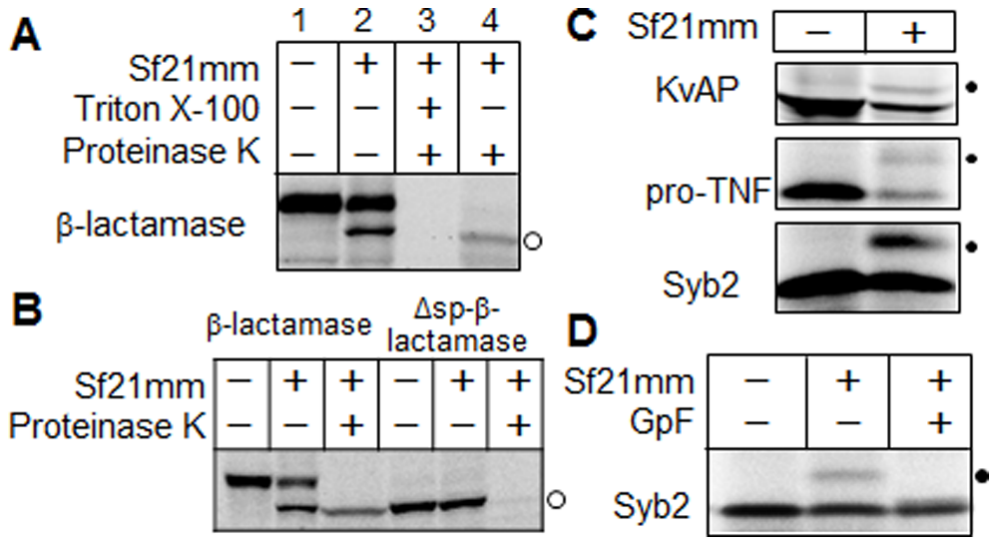
Assessment of posttranslational modification of secreted proteins and membrane proteins synthesized using the cell-free system containing Sf21 microsomes. (A) Cleavage of the signal peptide of β-lactamase in microsomes. Translocation reactions containing *E. coli* β-lactamase labeled with FluoroTect Green_Lys_ tRNA (12.5 µl) were mixed with 1 µl of sterilized water (lane 2), with 0.5 µl of 200 µg/ml proteinase K and 0.5 µl of 20% (v/v) Triton X-100 (lane 3) or 0.5 µl of 200 mg/ml proteinase K (lane 4). The samples were incubated for 1 h at 4°C. Equal volumes (6 µl) of the samples were separated by SDS-PAGE on 15% gels. The open circle marks the mature protein without the signal peptide. Lane 1 contains β-lactamase synthesized without microsomes as a control. (B) The N-terminal signal peptide sequence of β-lactamase is required for its translocation across the microsomal membrane. The *E. coli* β-lactamase lacking its signal peptide (Δsp-β-lactamase) and the wild type (β-lactamase) were labeled with FluoroTect Green_Lys_ tRNA (12.5 µl). The translocation reaction was performed as in panel A. (C) N-linked glycosylation of membrane proteins in the microsomes. *Aeropyrum pernix* voltage-dependent K^+^ channel (KvAP), human pro-tumor necrosis factor (pro-TNF) and human synaptobrevin II (Syb2) were labelled with ^35^S-methionine during translation in the presence (+) or absence (-) of Sf21 microsomes. Dots indicate the glycosylated form of the proteins. (D) Confirmation of N-linked glycosylation of Syb2 labeled with ^35^S-methionine. One µl of 10% (v/v) Triton X-100 and 1 µl of 500 mU/ml glycopeptidase F (GpF) were added to the translation mixture (12.5 µl), followed by incubation for 5 min at 37°C. The dot indicates the glycosylated protein.

The suitability of Sf21 microsomes for cell-free protein translocation was assessed using a range of different proteins. Functioning of signal peptide cleavage by Sf21 microsomes was examined using a secreted protein, *β*-lactamase (Fig. 2A). After addition of Sf21 microsomes, a smaller-sized protein band appeared on the SDS-PAGE gel, indicating successful cleavage by signal peptidase and translocation of the protein into the Sf21 microsomes. The size of the smaller-sized band was identical to the size of *β*-lactamase lacking the N-terminal signal peptide (Δsp-*β*-lactamase, Figure 2B), confirming that the microsomes contained the proper processing activity for secreted proteins. This was further supported by the finding that the lower band was resistant to exogenously added proteinase K, whereas the upper band, representing uncleaved *β*-lactamase, was readily hydrolyzed (Figs. 2A and 2B). In the presence of Triton X-100 both bands were completely digested by proteinase K (Fig. 2A). Next, *N*-linked glycosylation activity of the Sf21 microsomes was tested with the voltage-dependent K^+^ channel KvAP, a polytopic membrane protein, the pro-tumor necrosis factor pro-TNF, a membrane anchored protein, and the TA-protein synaptobrevin II (Syb2) (Figs. 2C and 2D). In the presence of Sf21 microsomes an additional higher band, corresponding to the *N-*glycosylated form of the protein, appeared in each case (Fig. 2D). When glycopeptidase F was added to the Syb2 protein preparation the higher band disappeared, indicating that it was indeed the N-glycan of Syb2 (Fig. 2D) and confirming that the Sf21 microsomes possessed *N*-linked glycosylation activity. These data show that the microsomes contained both the translocon and the GET system.

**Figure 3:**
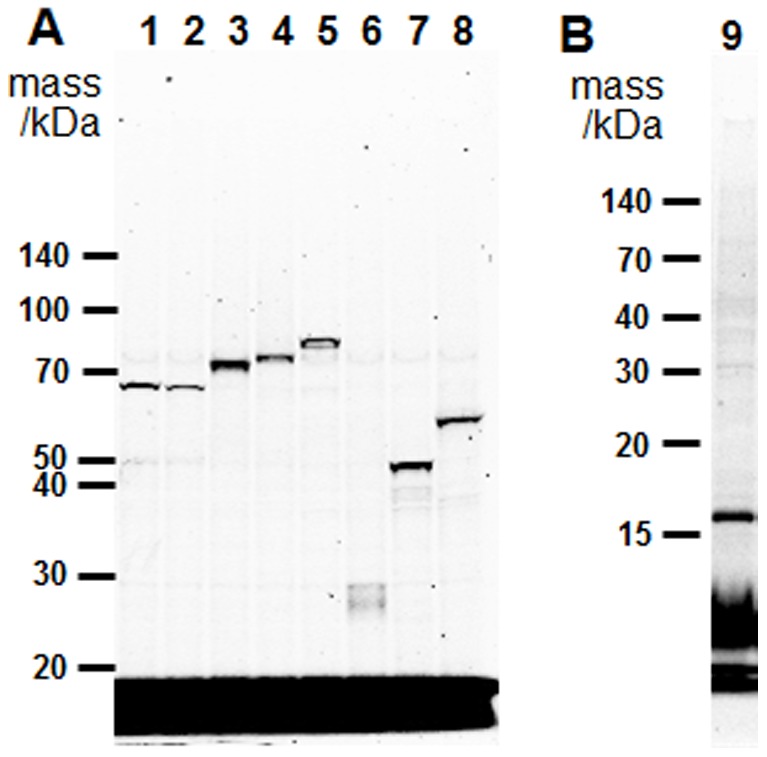
Production of different membrane proteins using the cell-free system. After synthesis of the membrane proteins in the cell-free system in the presence of FluoroTect Green_Lys_ tRNA, the membrane fraction was precipitated and separated by SDS/PAGE. (A) 10% polyacrylamide gel; lane 1, AtKC1 (plant, K^+^ channel, 6 transmembrane segments (TMS)); lane 2, KAT1 (plant, K^+^ channel, 6 TMS); lane 3, KAT2 (plant, K^+^ channel, 6 TMS); lane 4, GORK (plant, K^+^ channel, 6 TMS); lane 5, AKT1 (plant, K^+^ channel, 6 TMS); lane 6, NhaA (bacteria, Na^+^/H^+^ antiporter, 10 TMS); Lane 7, AspT (bacteria, aspartate: alanine antiporter, 10 TMS); lane 8, MsbA (bacteria, ABC transporter, 12 TMS). (B) 15% polyacrylamide gel; lane 9, Sec61 β(human, 1 TMS).

Integration of proteins with one to twelve membrane-spanning domains from various organisms in addition to that of KvAP shown in Fig. 2 was also tested using the cell-free system. In order to enable detection of the proteins FluoroTect Green_Lys_ tRNA was included in the reactions (Fig. 3). Both membrane-embedded and membrane-anchored proteins could be seen as a single band in the microsomes. The translation products were not detected in the supernatant fraction (data not shown). Thus Sf21 microsomes were a powerful tool for the membrane integration of a wide range of membrane proteins.

### Purification and reconstitution of a cell-free synthesized aspartate transporter

**Figure 4:**
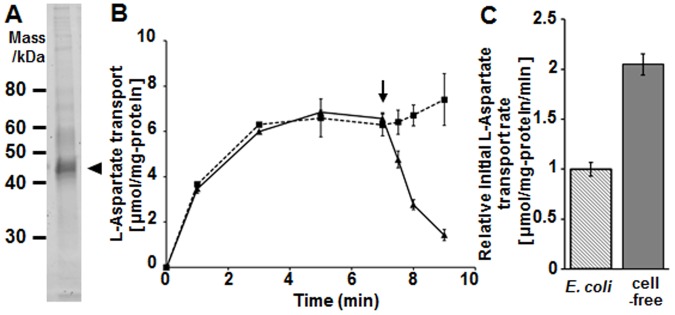
Reconstitution of the AspT aspartate transporter synthesized in the cell-free system with Sf21 microsomes. (A) Cell-free synthesized AspT purified with cobalt affinity resin under non-denaturing conditions was solubilized with 1.5% DDM, and eluted with 0.01% DDM and 250 mM imidazole. The purified AspT protein (arrow head) was subjected to SDS-PAGE. (B) L-aspartate transport activity of proteoliposomes containing purified AspT protein. Proteoliposomes loaded with 100 mM L-aspartate were resuspended in 50 mM phosphate buffer (pH 7) (8.3 µg protein/ml). At 0 min, L-[^3^H] aspartate was added into the buffer (2.5 mM final concentration). After the rate of influx and efflux of L-[^3^H] aspartate was equal (at steady state), non-radiolabelled L-aspartate was added to a final concentration of 15 mM at 7.5 min (solid line, arrow indicating time of addition of unlabelled substrate). The broken line corresponds to the same experiment performed without addition of unlabelled L-aspartate. (C) Comparison of the initial uptake rates for L-aspartate into *E. coli* expressing AspT (hatched bar) and into microsomes containing cell-free synthesized AspT (solid bar). The transport activity at 1 min was regarded as the initial uptake rate.

In order to expand upon the applications of the cell-free system, the bacterial aspartate transporter (AspT) was purified from Sf21 microsomes using a TALON affinity column and functionally reconstituted into non-ionic detergent DDM micelles (Fig. 4A). From 10 ml of initial reaction volume 3,69 µg of AspT were obtained. The proteoliposomes containing purified AspT accumulated L-[^3^H]aspartate after the addition of L-[^3^H]aspartate into the external buffer until 7 min (Fig. 4B). At 7 min, addition of an excess of unlabeled L-aspartate led to the release of L-[^3^H]aspartate (solid line), which represented AspT-mediated aspartate exchange activity. In the control (broken line) without addition of unlabeled L-aspartate at 7 min the concentrations of L-[^3^H]aspartate in the proteoliposomes did not decrease. Reconstituted AspT showed steady-state transport of L-(^3^H)-aspartate at approximately 6 µmol/mg of protein (Fig. 4B). To evaluate the transport activity of AspT synthesized in the cell-free system it was compared to the activity of AspT produced in *E. coli* [25]. Interestingly, AspT synthesized in the cell-free system had about two-fold higher initial aspartate transport rate than AspT expressed in *E. coli* (Fig. 4C).

### Incorporation of fluorescent non-natural amino acids by tRNAs recognizing the amber codon or a four-base codon into membrane proteins

**Figure 5:**
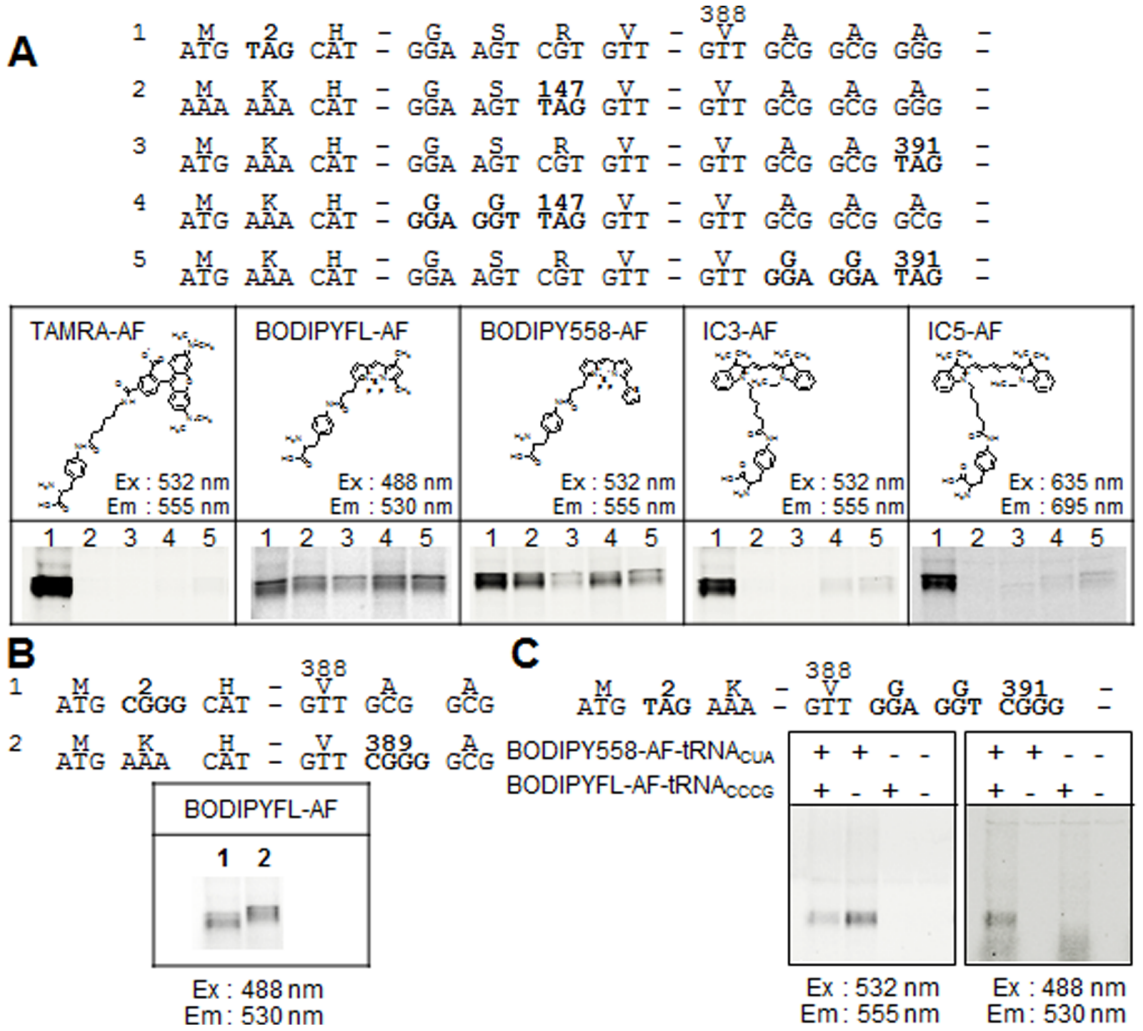
Incorporation of fluorescent non-natural amino acids into NhaA. (A) Five version of NhaA (1–5) with introduced amber (TAG) codons (or Gly-Gly-TAG, introduced changes in bold) that enable incorporation of fluorescent non-natural amino acids into the N-terminal, middle or C-terminal regions of the protein (top panel) were expressed in the insect cell-free translation and translocation system in the presence of different fluorescent non-natural amino acids. The valine (V388) at the C-terminal end of NhaA was fused to additional sequences in the linker of the vectors (Table 1). Labeled NhaA protein was subjected to SDS-PAGE and fluorescent images were taken at the indicated excitation and emission wavelengths (bottom panel). The structure of the different fluorescent non-natural amino acids used in the experiments is shown above the gel images. (B) Incorporation of fluorescent non-natural amino acids into N-terminal or C-terminal positions in NhaA at an introduced four-base codon (CGGG) (top panel). NhaA was synthesized using the cell-free translation and translocation system in the presence of BODIPYFL-AF, subjected to SDS-PAGE and fluorescent images taken at the indicated excitation and emission wavelengths (bottom panel). (C) Double-labeling of NhaA by incorporation of BODIPY558-AF at an amber codon introduced into the N-terminal region and BODIPYFL-AF at the CGGG four-base codon introduced into the C-terminal region (top panel). NhaA was synthesized using the cell-free translation and translocation system in the presence of one or both fluorescent amino acids. Images of the SDS-PAGE were taken at the two different excitation and emission wavelengths indicated (bottom panel).

To test the incorporation of non-natural amino acids into proteins synthesized in the cell-free system, two different types of suppressor amino-acylated tRNA [22], [23], which recognize the amber codon (TAG) and a four-base codon (CGGG), respectively, were added to the reaction. We chose the 12 membrane-spanning protein NhaA as a representative membrane protein and inserted either the amber codon or the four-base codon into its cytoplasmic N-terminal, middle or C-terminal region (Fig. 5). In all cases fluorescence was highest when the inserted codon was in the N-terminal region instead of in the middle or C-terminal positions. BODIPY-amino-phenylalanine (AF) and BODIPY558-AF, which have smaller side chains, were more readily incorporated into positions in the middle or C-terminal positions than TAMRA-AF, IC3-AF and IC5-AF, which have larger side chains (lanes 1–3). Replacing one or two amino acids before the inserted amber codon with glycine (lanes 4 and 5) increased the intensity of the labeling, possibly because the glycine residues reduced local steric hindrance and enhanced the incorporation of the fluorescent non-natural amino acids.

BODIPYFL-AF was incorporated by the four-base-codon-recognizing t-RNA into the N-terminal and the C-terminal region of NhaA to a similar extent (Fig. 5B). The difference in the mobility of the two proteins is most likely an effect of the different non-natural amino acids integrated at the two positions. Next we tested double labeling of NhaA containing the amber codon in the N-terminal and Gly-Gly followed by the four-base codon for BODIPYFL-AF in the C-terminal region. BODIPY558-AF and BODIPYFL-AF as well as the corresponding tRNAs recognizing the amber codon and CGGG, respectively, were added to the reaction. Both non-natural amino acids were incorporated into NhaA (Fig. 5C). In the absence of BODIPY558-AF-tRNA_CUA_, no protein was made (due to the stop codon at position 2). In contrast, even when no BODIPYFL-AF-tRNA_CCCG_ was added, the full length NhaA was produced.

### Labeling of membrane proteins with non-natural amino acids inside and outside of the microsomes

**Figure 6:**
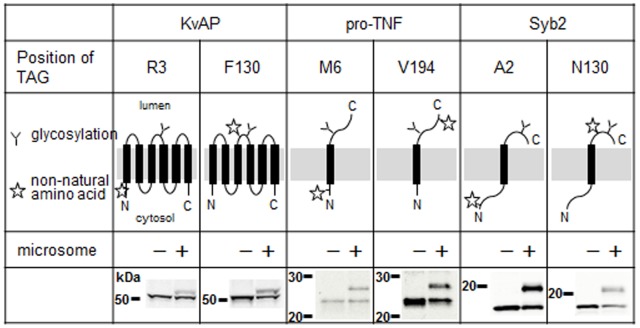
Position-specific incorporation of fluorescent amino acids by the cell-free system. RNA templates encoding variants of KvAP, pro-TNF, or Syb2 were translated in the cell-free system supplemented with BODIPYFL-AF conjugated tRNA with or without the addition of Sf21microsomes (microsomes). The non-natural amino acids were incorporated into introduced TAG codon. The resulting proteins were subjected to SDS-PAGE (10-15% polyacrylamide) and detected with excitation at 488 nm and emission at 530 nm. The amber codon in the different constructs was substituted for the codon at the position corresponding to the listed amino acids (except for M6 in pro-TNF where the TAG codon was inserted as an additional codon after the M6 codon) and marked by a star in the diagram.

The results in Fig. 5A showed that Sf21 microsomes were capable of incorporation of non-natural amino acids into intracellular regions of NhaA. However, it is possible that the relatively large side chain of fluorescent non-natural amino acids could impair translocation of these amino acids across the ER membrane of the Sf21 microsomes. To test whether labeling at positions in extracellular domains can be obtained with Sf21 microsomes, new amber codons were introduced into constructs for KvAP, pro-TNF and Syb2 (see Fig. 2B), either into an intracellular or an extracellular part of the protein. The incorporation of non-natural amino acid was mediated by BODIPYFL-AF-tRNA_CUA_ in the cell-free system (Fig. 6). All three proteins showed bands of similar intensity when the fluorescent amino acid was incorporated at the extracellular side (upper band). These results indicated that BODIPYFL-AF was able to efficiently cross the ER membrane via both the translocon and the GET system.

## Discussion

Rough microsomes of consistent quality are essential for the reproducible cell-free production of membrane proteins. We isolated rough microsomes from Sf21 insect cell suspension cultures and demonstrated that they were of sufficient quality to produce functional membrane proteins. The isolated microsomes not only enabled the cotranslational insertion of SRP-dependent membrane proteins but also the posttranslational integration of TA-proteins into the ER membrane. Because of their high translational activity, Sf21 insect cell cultures are well established as a host for the overexpression of foreign proteins via the Baculovirus expression system. Sf21 cultured insect cells can be maintained as stable suspension cultures. Cultured cells also have the advantage that it is easy to scale up the size of experiments and that extracts of a consistent nature can be more readily obtained than from cells isolated from harvested organs (e.g. dog pancreas). Due to these advantages, a cell-free translation system isolated from cultured insect cells had been previously developed [10]. As shown in our study, microsomes extracted from the same source provided reliable and uniform material for the cell-free co- and post-translational translocation of membrane proteins.

The preparation of rough microsomes from Sf21 cultured cells (Sf21 microsomes) was performed by a modified protocol based on the method developed for the isolation of dog pancreas rough microsomes [1], [13], [14] (Fig. 1). Due to the different starting material several steps were not required during the isolation of microsomes from cultured cells. In the wheat germ system, column washing of the microsomes is required because the high-ionic-strength buffer, in which the rough microsomes are resuspended, removes the SRP from the membranes. In our insect cell protocol no high ionic strength buffer was used and therefore column washing was not necessary. In preparing dog pancreas rough microsomes EDTA-stripping and nuclease treatment are performed to reduce the background in the subsequent translation and translocation reactions [13] but these steps could be omitted in our procedure. Our simplified protocol improved the performance of the cell-free system probably because it reduced the loss of cellular components required for successful cotranslational integration of membrane proteins (e.g. SRP) by the translocon and the GET-system.

One possible application of a cell-free translation/translocation system is to label a target protein with non-natural amino acids, which is more challenging when expressing a protein *in vivo*. We tested two methods for incorporation of artificial amino acids [28], [29] (Figs. 5 and 6). Application of tRNAs for the amber codon or for the recognition of a four–base codon both resulted in the incorporation of non-natural amino acids into proteins with one, six or twelve membrane-spanning domains. It was even possible to obtain double labeling with different BODIPY fluorophores within a single protein with twelve membrane-spanning domains, NhaA (Fig. 5C). This application may therefore be useful for applications relying on the ability to detect fluorescent proteins, for instance for the analysis of intramolecular interaction by fluorescence resonance energy transfer (FRET). One caveat is that the size of the side chain of the non-natural amino acids may restrict both translation and translocation processes. Specifically, we predicted that this would be more of a problem when the labeling amino acid was introduced at the extracellular side of the protein. However, in our example BODIPY-FL was efficiently incorporated on both the cytosolic side as well as on the lumen side of three different membrane proteins inserted either via the translocon-mediated system or the GET system (Figs. 5 and 6).

Cell-free synthesis is a promising tool to achieve a high yield of cytotoxic membrane proteins that do not readily accumulate in living cells. Production of *Lactobacillus* aspartate transporter AspT by heterologous expression in *E. coli* causes retarded growth [30]. Here we were able to obtain sufficient amounts of AspT from the cell-free system and to successfully reconstitute it into a lipid bilayer (Fig. 4). Subsequent characterization of the transport activity of the reconstituted AspT demonstrated that its activity was comparable to that of the same protein purified from *E. coli*.

Less than ten percent of membrane proteins are TA-proteins, which possess a single C-terminal transmembrane helix and a cytosol-facing N-terminal region [2]. Due to these topological characteristics, these proteins must be targeted and inserted into the ER membrane by an SRP-independent posttranslational pathway. Recent studies showed that the posttranslational insertion of TA-proteins into the ER membrane requires the cooperation of the ER-to-Golgi trafficking complex (GET; guided entry of TA proteins pathway in yeast), which contains Get 1-5 [31], [32]. The GET complex was functional in our cell-free system (Figs. 2 and 3 and 6), indicating that the cell-free system isolated from cultured insect cells is capable of carrying out the biogenesis of TA-proteins in the ER membrane.

The data presented here show that microsomes are a powerful ER membrane system for cell-free membrane protein synthesis. It is to be expected that this method can also be adapted to the use of microsomes from other cultured cells. By using cultured cells as a source material for homogenous microsomes, it should be possible to successfully express cytotoxic membrane proteins. Furthermore, it is feasible that the system described here could be made available as a kit for a wider audience interested in producing membrane proteins more easily.

## Supporting Information

S1 Figure **SDS-polyacrylamide gel electrophoresis (12.5%) of 2 µl of the insect cell extract used for cell-free translation (lysate) and 0.4 µl of the microsomes (Sf21mm).** The insect cell extract was prepared as described previously [10].
(PPTX)

## References

[1] Dultz E, Hildenbeutel M, Martoglio B, Hochman J, Dobberstein B, et al (2008) The signal peptide of the mouse mammary tumor virus Rem protein is released from the endoplasmic reticulum membrane and accumulates in nucleoli. Journal of Biological Chemistry 283:9966–9976. 18270201 10.1074/jbc.M705712200

[2] Shao SC, Hegde RS (2011) Membrane protein insertion at the endoplasmic reticulum. Annual Review of Cell and Developmental Biology, Vol 27 27:25–56. 10.1146/annurev-cellbio-092910-154125 PMC4163802 21801011

[3] Chen HZ, Zubay G (1983) Prokaryotic coupled transcription translation. Methods in Enzymology 101:674–690. 6310341 10.1016/0076-6879(83)01047-2

[4] Shimizu Y, Inoue A, Tomari Y, Suzuki T, Yokogawa T, et al (2001) Cell-free translation reconstituted with purified components. Nature Biotechnology 19:751–755. 10.1038/90802 11479568

[5] Gasior E, Herrera F, Sadnik I, Mclaughlin CS, Moldave K (1979) Preparation and characterization of a cell-free system from *Saccharomyces-cerevisiae* that translates natural messenger ribonucleic-acid. Journal of Biological Chemistry 254:3965–3969. 374404

[6] Erickson AH, Blobel G (1983) Cell-free translation of messenger-rna in a wheat-germ system. Methods in Enzymology 96:38–50. 6656637 10.1016/s0076-6879(83)96007-x

[7] Sawasaki T, Hasegawa Y, Tsuchimochi M, Kamura N, Ogasawara T, et al (2002) A bilayer cell-free protein synthesis system for high-throughput screening of gene products. Febs Letters 514:102–105. 11904190 10.1016/s0014-5793(02)02329-3

[8] Jackson RJ, Hunt T (1983) Preparation and use of nuclease-treated rabbit reticulocyte lysates for the translation of eukaryotic messenger-RNA. Methods in Enzymology 96:50–74. 6656641 10.1016/s0076-6879(83)96008-1

[9] Swerdel MR, Fallon AM (1989) Cell-free translation in lysates from *Spodoptera-frugiperda* (Lepidoptera, Noctuidae) cells. Comparative Biochemistry and Physiology B-Biochemistry & Molecular Biology 93:803–806. 10.1016/0305-0491(89)90049-7 2805641

[10] Ezure T, Suzuki T, Higashide S, Shintani E, Endo K, et al (2006) Cell-free protein synthesis system prepared from insect cells by freeze-thawing. Biotechnology Progress 22:1570–1577. 17137303 10.1021/bp060110v

[11] Blobel G, Dobberstein B (1975) Transfer of proteins across membranes.2. reconstitution of functional rough microsomes from heterologous components. Journal of Cell Biology 67:852–862. 811672 10.1083/jcb.67.3.852 PMC2111655

[12] Lundin C, Kall L, Kreher SA, Kapp K, Sonnhammer EL, et al (2007) Membrane topology of the *Drosophila* OR83b odorant receptor. Febs Letters 581:5601–5604. 18005664 10.1016/j.febslet.2007.11.007 PMC2176074

[13] Walter P, Blobel G (1983) Preparation of microsomal-membranes for cotranslational protein translocation. Methods in Enzymology 96:84–93. 6656655 10.1016/s0076-6879(83)96010-x

[14] Scheele G (1983) Methods for the study of protein translocation across the rer membrane using the reticulocyte lysate translation system and canine pancreatic microsomal-membranes. Methods in Enzymology 96:94–111. 6656656 10.1016/s0076-6879(83)96011-1

[15] Utsumi T, Akimaru K, Kawabata Z, Levitan A, Tokunaga T, et al (1995) Human pro-tumor necrosis factor - molecular determinants of membrane translocation, sorting, and maturation. Molecular and Cellular Biology 15:6398–6405. 7565792 10.1128/mcb.15.11.6398 PMC230891

[16] Ezure T, Suzuki T, Shikata M, Ito M, Ando E, et al (2007) Expression of proteins containing disulfide bonds in an insect cell-free system and confirmation of their arrangements by MALDI-TOF MS. Proteomics 7:4424–4434. 18072203 10.1002/pmic.200700774

[17] Whitley P, Grahn E, Kutay U, Rapoport TA, vonHeijne G (1996) A 12-residue-long polyleucine tail is sufficient to anchor synaptobrevin to the endoplasmic reticulum membrane. Journal of Biological Chemistry 271:7583–7586. 8631791 10.1074/jbc.271.13.7583

[18] Ota K, Sakaguchi M, Hamasaki N, Mihara K (1998) Assessment of topogenic functions of anticipated transmembrane segments of human band 3. Journal of Biological Chemistry 273:28286–28291. 9774451 10.1074/jbc.273.43.28286

[19] Sato Y, Sakaguchi M, Goshima S, Nakamura T, Uozumi N (2002) Integration of shaker-type K^+^ channel, KAT1, into the endoplasmic reticulum membrane: Synergistic insertion of voltage-sensing segments, S3–S4, and independent insertion of pore-forming segments, S5-P-S6. Proceedings of the National Academy of Sciences of the United States of America 99:60–65. 11756658 10.1073/pnas.012399799 PMC117514

[20] Utsumi T, Ohta H, Kayano Y, Sakurai N, Ozoe Y (2005) The N-terminus of B96Bom, a Bombyx mori G-protein-coupled receptor, is N-myristoylated and translocated across the membrane. Febs Journal 272:472–481. 15654885 10.1111/j.1742-4658.2004.04487.x

[21] Moriya K, Nagatoshi K, Noriyasu Y, Okamura T, Takamitsu E, et al**.** (2013) Protein N-myristoylation plays a critical role in the endoplasmic reticulum morphological change induced by overexpression of protein lunapark, an integral membrane protein of the endoplasmic reticulum. PLoS One 8. 10.1371/journal.pone.0078235 PMC3817238 24223779

[22] Taki M, Tokuda Y, Ohtsuki T, Sisido M (2006) Design of carrier tRNAs and selection of four-base codons for efficient incorporation of various nonnatural amino acids into proteins in *Spodoptera frugiperda* 21 (Sf21) insect cell-free translation system. Journal of Bioscience and Bioengineering 102:511–517. 17270715 10.1263/jbb.102.511

[23] Kajihara D, Abe R, Iijima I, Komiyama C, Sisido M, et al (2006) FRET analysis of protein conformational change through position-specific incorporation of fluorescent amino acids. Nature Methods 3:923–929. 17060916 10.1038/nmeth945

[24] Sato Y, Aizawa K, Ezure T, Ando E, Uozumi N (2012) A simple fed-batch method for transcription and insect cell-free translation. Journal of Bioscience and Bioengineering 114:677–679. 22841867 10.1016/j.jbiosc.2012.06.015

[25] Nanatani K, Fujiki T, Kanou K, Takeda-Shitaka M, Umeyama H, et al (2007) Topology of AspT, the aspartate: alanine antiporter of *Tetragenococcus halophilus*, determined by site-directed fluorescence labeling. Journal of Bacteriology 189:7089–7097. 17660287 10.1128/JB.00088-07 PMC2045216

[26] Nanatani K, Maloney PC, Abe K (2009) Structural and functional Importance of transmembrane domain 3 (TM3) in the aspartate: alanine antiporter AspT: Topology and function of the residues of TM3 and oligomerization of AspT. Journal of Bacteriology 191:2122–2132. 19181816 10.1128/JB.00830-08 PMC2655511

[27] Sasahara A, Nanatani K, Enomoto M, Kuwahara S, Abe K (2011) Substrate specificity of the aspartate: alanine antiporter (AspT) of *Tetragenococcus halophilus* in reconstituted liposomes. Journal of Biological Chemistry 286:29044–29052. 21719707 10.1074/jbc.M111.260224 PMC3190712

[28] Noren CJ, Anthonycahill SJ, Griffith MC, Schultz PG (1989) A general-method for site-specific incorporation of unnatural amino-acids into proteins. Science 244:182–188. 2649980 10.1126/science.2649980

[29] Hohsaka T, Kajihara D, Ashizuka Y, Murakami H, Sisido M (1999) Efficient incorporation of nonnatural amino acids with large aromatic groups into streptavidin in in vitro protein synthesizing systems. Journal of the American Chemical Society 121:34–40.

[30] Nanatani K, Ohonishi F, Yoneyama H, Nakajima T, Abe K (2005) Membrane topology of the electrogenic aspartate-alanine antiporter AspT of *Tetragenococcus halophilus* . Biochemical and Biophysical Research Communications 328:20–26. 15670744 10.1016/j.bbrc.2004.12.133

[31] Yamamoto Y, Sakisaka T (2012) Molecular machinery for insertion of tail-anchored membrane proteins into the endoplasmic reticulum membrane in mammalian cells. Molecular Cell 48:387–397. 23041287 10.1016/j.molcel.2012.08.028

[32] Denic V, Dotsch V, Sinning I (2013) Endoplasmic reticulum targeting and Insertion of tail-anchored membrane proteins by the GET pathway. Cold Spring Harbor Perspectives in Biology 5. 10.1101/cshperspect.a013334 PMC3721280 23906715

