# Supplementary figures and images for: A Cell-Free Translocation System Using Extracts of Cultured Insect Cells to Yield Functional Membrane Proteins
Source: PLoS One. 2014 Dec 8;9(12):e112874. doi: 10.1371/journal.pone.0112874 (PMC4259328; doi:10.1371/journal.pone.0112874)

## Slide 1
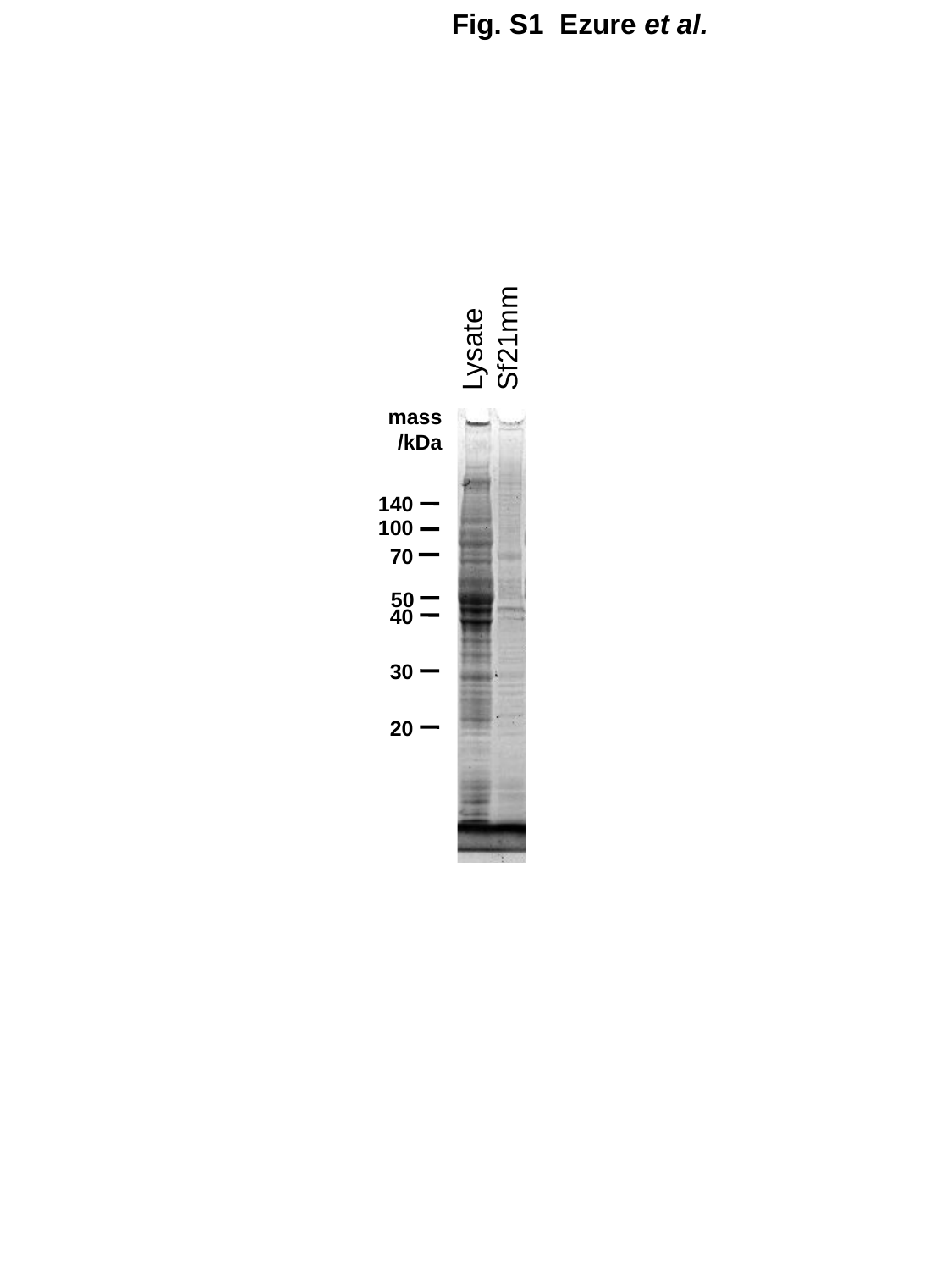

Fig. S1 Ezure et al.
Lysate
Sf21mm
mass
/kDa
140
100
70
50
40
30
20

Supplement: S1 Figure — SDS-polyacrylamide gel electrophoresis (12.5%) of 2 µl of the insect cell extract used for cell-free translation (lysate) and 0.4 µl of the microsomes (Sf21mm). The insect cell extract was prepared as described previously [10]. (PPTX) [file pone.0112874.s001.pptx]
